# Vector characterization of zero-order terahertz Bessel beams with linear and circular polarizations

**DOI:** 10.1038/s41598-017-12524-y

**Published:** 2017-10-24

**Authors:** Zhen Wu, Xinke Wang, Wenfeng Sun, Shengfei Feng, Peng Han, Jiasheng Ye, Yan Zhang

**Affiliations:** Beijing Key Laboratory of Metamaterials and Devices, Beijing Advanced Innovation Center for Imaging Technology, Key Laboratory of Terahertz Optoelectronics, Ministry of Education, and Department of Physics, Capital Normal University, Beijing, 100048 P.R. China

## Abstract

As a kind of special beams, Bessel beams are always a research hot spot in optics due to its non-diffractive and self-healing properties. Here, zero-order terahertz (THz) Bessel beams with linear and circular polarizations are generated by using a THz quarter wave plate and Teflon axicons with different opening angles. By applying a THz digital holographic imaging system, the evolutions of the transverse (*E*_*x*_, *E*_*y*_) and longitudinal (*E*_*z*_) electric fields are coherently measured and analyzed during the propagation processes of the THz Bessel beams. The vectorial Rayleigh diffraction integral is used to accurately reproduce the amplitude, phase, and non-diffractive feature of each polarization component for the THz Bessel beams. With varying opening angles of the axicons, the focal spots, diffraction-free ranges, and Gouy phase shifts of the THz Bessel beams are compared and discussed. The experiment and simulation results provide a comprehensive view for exactly understanding peculiar features of THz Bessel beams.

## Introduction

The concept of a non-diffractive Bessel beam was firstly proposed by Durnin *et al*. in 1987^1^. Because this kind of optical beam can keep a constant intensity distribution in a certain propagation distance, they have strong application values in optical imaging^2^, optical fabrication^3^, particle acceleration^4^, and so on. Therefore, the investigation of Bessel beams is always an important exploring direction in optics. As a novel far-infrared radiation, terahertz (THz) wave and its special properties have been gradually known by the masses over the past decades^5^. With the THz technology maturing, it has shown strong application potentials, such as material identification^6^, security inspection^7^, non-invasive flaw detection^8^, and optical communications^9^.

Recently, investigations on the Bessel beams are becoming a hot spot in the THz field, which may push the development of current THz systems due to the special diffraction characteristics of this kind of optical beam. In 2009, Winnerl *et al*. attempted to use large-area microstructured photoconductive antennas to generate THz Bessel-Gauss beams with radial and azimuthal polarizations^10^. In 2012, Bitman *et al*. demonstrated that a pulsed THz Bessel beam can enhance the ability of obtaining depth information in a THz imaging system^11^. In 2015, Knyazev *et al*. utilized a free electron laser and silicon binary elements to form THz vortex Bessel beams with different topological charges and further excite THz surface plasmon polaritons on the edge of a metal-dielectric interface^12^. In 2015, Wei *et al*. applied a 3D printing technique to fabricate polymer spiral axicons with different topological numbers and generated high-order THz Bessel beams^13^. In 2016, Liu *et al*. experimentally achieved THz Bessel beams with normal and oblique propagation directions by using a transmission-type coding metasurface^14^. Obviously, researchers have paid more and more attention to the generation methods and application fields of the THz Bessel beams.

In this work, zero-order THz Bessel beams with linear and circular polarizations are generated by employing Teflon axicons with different opening angles and a THz quarter wave plate (TQWP). Vectorial properties of the THz Bessel beams are comprehensively observed by using a THz digital holographic imaging system, including the transverse (*E*_*x*_, *E*_*y*_) and longitudinal (*E*_*z*_) electric field components. By performing Z-scan measurements, non-diffractive features of the THz Bessel beams are recorded and compared, including the focal spot sizes, diffraction-free ranges, and Gouy phase shifts. The vectorial Rayleigh diffraction integral is adopted to simulate the experimental phenomena. This work vividly exhibits a full view of the propagation process of a THz Bessel beam.

## Experimental Design

**Figure 1:**
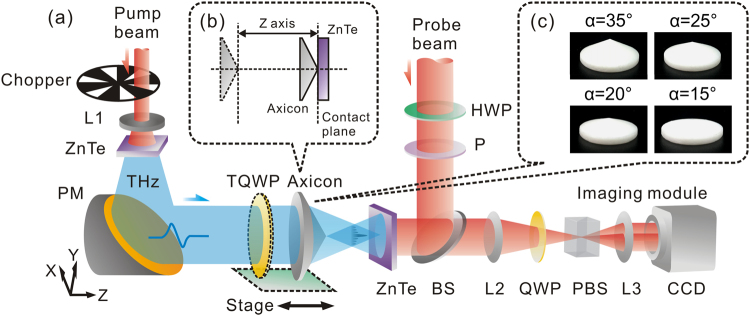
Experimental setup and samples. (**a**) THz digital holographic imaging system for characterizing vectorial properties of zero-order THz Bessel beams. (**b**) Shows the distance variation between the axicon and the detection crystal during the Z-scan measurement. The contact plane between the tip of the axicon and the detection crystal is considered as the original point. (**c**) Exhibits the photographs of Teflon aixcons with opening angles of 30°, 25°, 20°, 15°.

To observe vectorial properties of zero-order THz Bessel beams, a THz digital holographic imaging system is utilized as the testing platform, as shown in Fig. 1a. An x-polarized THz wave gets into a Teflon axicon to generate a zero-order THz Bessel beam. The transverse (*E*_*x*_, *E*_*y*_) and longitudinal (*E*_*z*_) components of the transmitted THz field are measured by using the imaging system, respectively^15,16^. To record the propagation process of the THz Bessel beam, the position of the axicon is linearly varied with respect to the measurement plane for fulfilling a Z-scan measurement. In the measurement, the contact plane between the tip of the axicon and the detection crystal is considered as the original point, as shown in Fig. 1b. The merit of the measurement mode is that the linear phase shift of the THz wave is effectively avoided, because the total optical path of the THz beam is not varied during the measurement. In addition, a TQWP is used to adjust the polarization of the incident THz wave. To compare discrepancies of THz Bessel beams with different non-diffractive zones, four commercial Teflon axicons with opening angles of 30°, 25°, 20°, 15° and an aperture diameter of 19 mm are selected as the test samples, as shown in Fig. 1c.

## Results and Discussion

### Vectorial properties of linearly polarized THz Bessel beams

**Figure 2:**
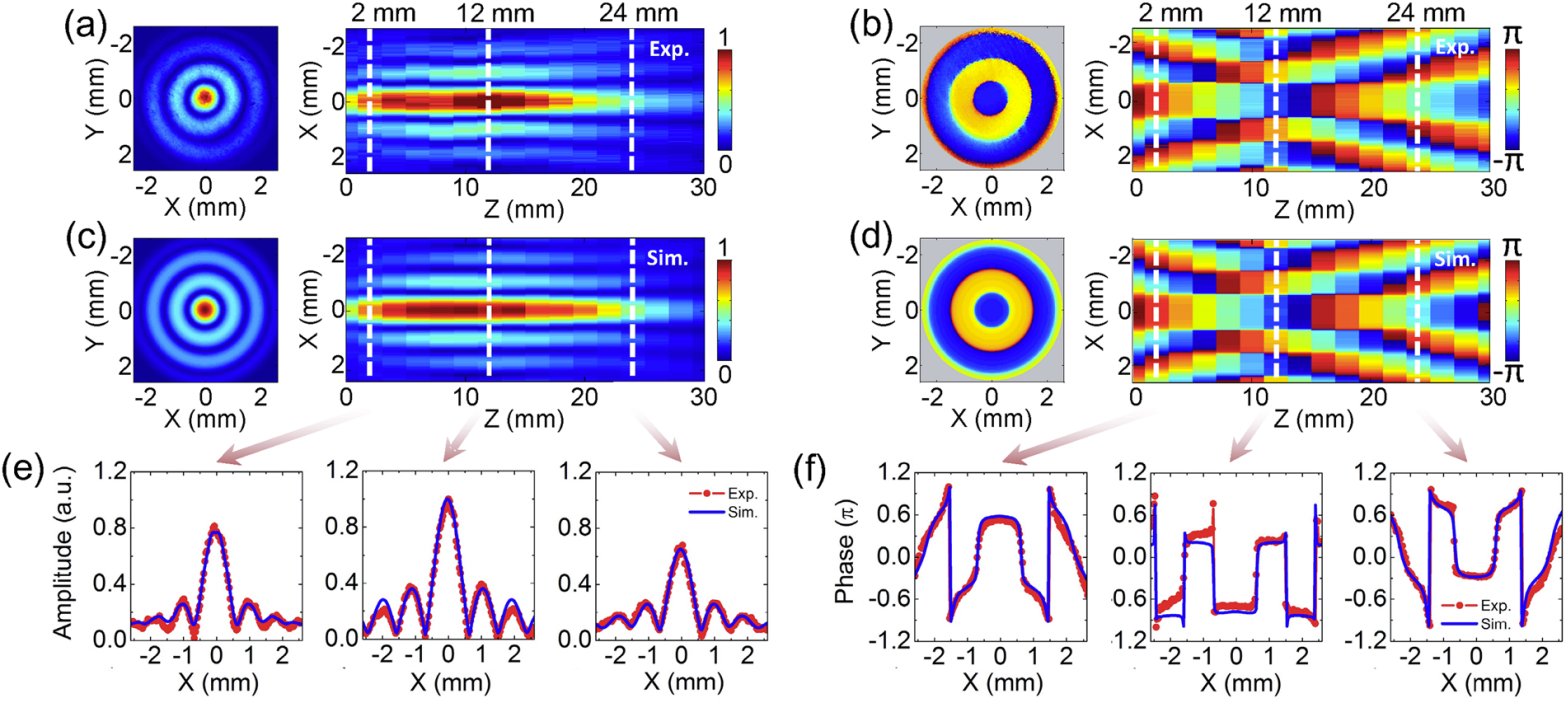
*E*_*x*_ component of a linearly polarized THz Bessel beam generated by using a Teflon axicon with an opening angle of 30°. (**a**) Shows a transverse amplitude distribution of *E*_*x*_ with 0.6 THz at 12 mm and a longitudinal amplitude pattern on the cross-section of y = 0 mm, respectively. (**b**) Presents the corresponding transverse and longitudinal phase patterns. (**c**,**d**) Are the simulated amplitude and phase patterns on the x-y and x-z planes by utilizing a vectorial Rayleigh diffraction integral. (**e**,**f**) Shows the transverse amplitude and phase profiles extracted from the experiment and simulation results at z = 2 mm, 12 mm, and 24 mm. Their positions are marked by white dashed lines in (**a**–**d**).

First of all, the axicon with a 30° opening angle is measured with a 30 mm scan range and a 2 mm scan resolution. Figure 2a presents the amplitude distribution of the *E*_*x*_ component with 0.6 THz at z = 12 mm for the generated x-polarized THz Bessel beam and its corresponding longitudinal amplitude profile on the x-z plane (y = 0 mm). The THz field presents a circular central peak with a 1.4 mm diameter as well as two concentric ring side-lobes with 2.2 mm and 4.0 mm diameters. Its longitudinal cross section shows a propagation invariance in the range from z = 0 mm to 24 mm. The axially symmetric field pattern and the non-diffraction phenomenon of the THz beam are consistent with typical properties of a Bessel beam. According to ref.^2^, the radius *R* of the central peak and the diffraction-free range *Z*_*max*_ can be expressed as

1 $$R=\frac{2.4048\lambda }{2\pi (n-1)\tan \,\alpha },$$

2 $${Z}_{\max }=\frac{{\omega }_{0}}{\tan [\text{arc}\,\sin (n\,\sin \,\alpha )-\alpha ]},$$

where *α* is the opening angle of the axicon, *n* is the refractive index of the Teflon material, *ω*_0_ and *λ* are the radius and wavelength of the incident THz beam, respectively. In the experiment, these parameters are *α* = 30°, *n* = 1.426, *ω*_0_ = 7 mm, *λ* = 500 μm and the calculated results are 2 *R* = 1.55 mm as well as *Z*_*max*_ = 25.3 mm, which are basically in agreement with the experimental phenomena. The deviations between the experimental and calculated results are mainly ascribed to the fabrication error of the axicon. Here, it should be noted that *R* is defined as the distance from the center of the main peak to the first radial amplitude minimum and *Z*_*max*_ is defined as the full widths at half maximum of the THz amplitude along the optical axis^13^, respectively. To more accurately observe the non-diffractive feature of the THz Bessel beam, transverse amplitude profiles at z = 2 mm, 12 mm, and 24 mm are extracted and plotted in Fig. 2e. Their positions are marked by white dashed lines on the longitudinal amplitude cross section, as shown in Fig. 2a. It can be seen that the sizes of the central peaks and annular side-lobes are almost fixed in the propagation of the THz wave except for a degree of intensity variation. A merit of this imaging system is that the phase distribution of the THz field can be directly acquired due to the coherent detection. Figure 2b exhibits the wrapped phase map of the *E*_*x*_ component with 0.6 THz at z = 12 mm and the phase evolution process on the longitudinal cross section (y = 0 mm). It is apparent that the phase pattern is composed of several concentric circles. On each circular region, the phase value almost keeps constant, which results in the constructive interference of the THz wave. Besides, a phase jump of π occurs on the interface between adjacent circles, which leads to the destructive interference of the THz wave. Therefore, it is easily seen that the peculiar amplitude pattern of the THz Bessel beam arises from the characteristics of its phase. On the x-z plane, the phase distribution clearly manifests the interference effect of the THz beams refracted by the axicon. In the diffraction-free range, the phase always keeps a flat plane around the optical axis, which suggests that the THz field consistently maintains the constructive interference at different propagation distances. This is the reason causing the non-diffractive feature of the THz Bessel beam. Extracted transverse phase profiles at z = 2 mm, 12 mm, and 24 mm also clearly present a smooth distribution on the region of the central peak, as shown in Fig. 2f. In addition, the phase almost exhibits a linear variation on the optical axis with increasing the propagation distance, which is ascribed to the conic wave front of the THz Bessel beam. Taken together, the converging process of a Bessel beam differs from that of a Gaussian beam.

To further confirm the measurement accuracy, the vectorial Rayleigh diffraction integral is utilized to simulate the experimental phenomena. The *E*_*x*_ component of the THz Bessel beam can be expressed as^17,18^

3 $$\begin{array}{rcl}{E}_{x}(\rho ,\phi ,z) & = & \frac{-jz\pi \exp (jkr)}{\lambda {r}^{2}}\\  &  & \times {\int }_{0}^{l}d{\rho }_{0}\exp (\mu ){\rho }_{0}[({t}_{p}\,\cos \,\theta +{t}_{s}){J}_{0}(\eta )-\,({t}_{p}\,\cos \,\theta -{t}_{s}){J}_{2}(\eta )\cos \,2\phi ],\end{array}$$

where *k* is the wave number in vacuum, $(\rho ,\phi ,z)$ is the cylindrical coordinate on the observation plane, *t*_*p*_ and *t*_*s*_ are the Fresnel transmission coefficients through the oblique interface of the axicon for x- and y-polarized components, *l* is the aperture radius of the axicon, *θ* is the deflected angle with respect to the optical axis for the transmitted THz beam, which is induced by the axicon and can be expressed as $\theta =\text{arc}\,\sin (n\,\sin \,\alpha )-\alpha$. Here, $r=\sqrt{{\rho }^{2}+{z}^{2}}$, $\rho =\sqrt{{x}^{2}+{y}^{2}}$, and ${\rho }_{0}=\sqrt{{x}_{0}^{2}+{y}_{0}^{2}}$, where (*x*, *y*) and (*x*_0_, *y*_0_) are the Cartesian coordinates on the observation and incident planes. ${J}_{0}(\eta )$ and ${J}_{2}(\eta )$ are the Bessel functions of the first kind with the orders 0 and 2, respectively. *η* and *μ* can be expressed as

4 $$\eta =-\frac{k\rho {\rho }_{0}}{r},$$

5 $$\mu =[-\frac{{\rho }_{0}^{2}}{{\omega }_{0}^{2}}+jk\frac{{\rho }_{0}^{2}}{2r}-jk{\rho }_{0}(n-1)\tan \,\alpha ].$$

Figure 2c and d show the simulated amplitude and phase patterns on the x-y and x-z planes, which are in agreement with the experimental phenomena. For more intuitive comparison, transverse amplitude and phase profiles at z = 2 mm, 12 mm, and 24 mm are also extracted from Fig. 2c and d, as shown in Fig. 2e and f. The maximum divergence between simulated and experimental amplitude profile curves does not exceed 8%. On the phase profile curves, there are a little bit differences between experiment and simulation results on a few pixels, which are induced by some abrupt phase shifts due to the measurement noise.

**Figure 3:**
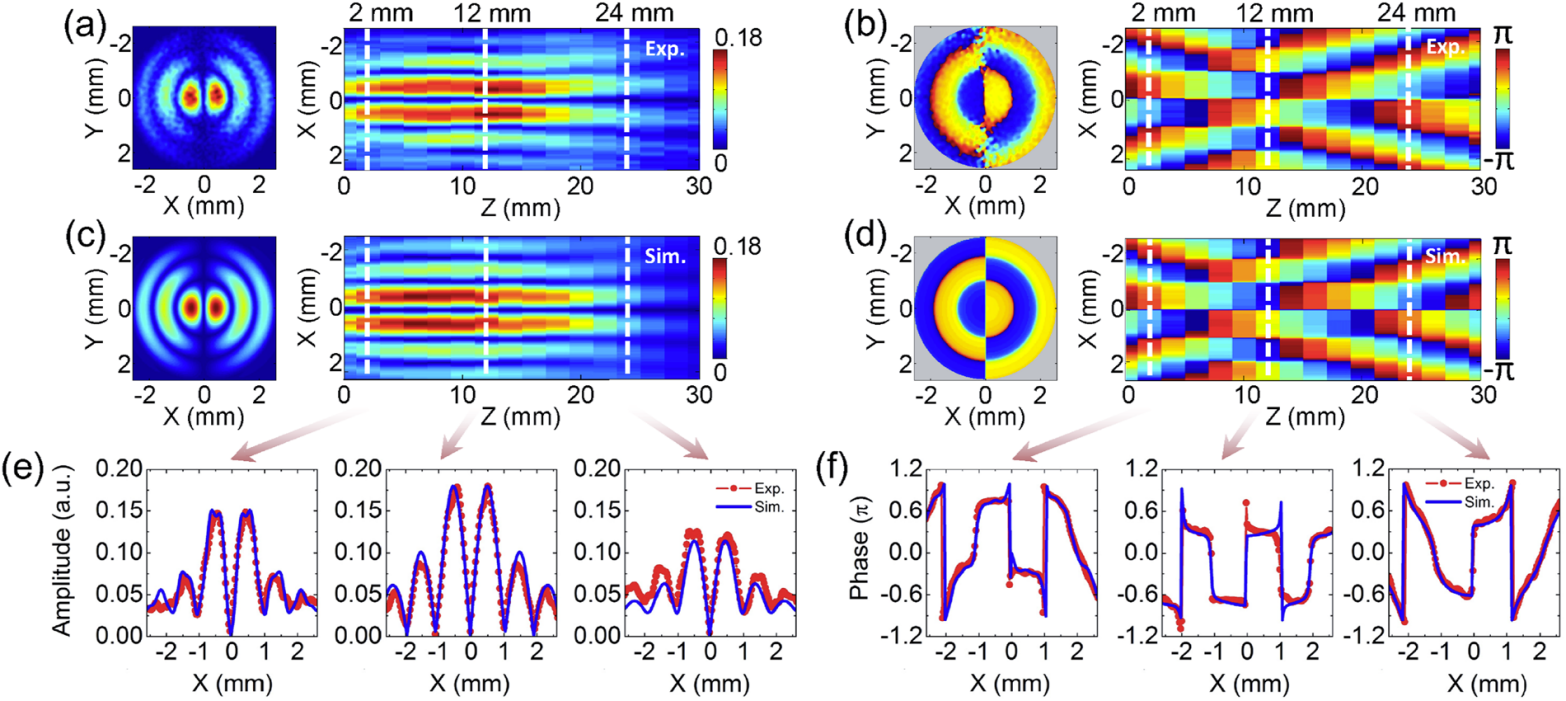
*E*_*z*_ component of the THz Bessel beam with a linear polarization. (**a**,**b**) Give transverse amplitude and phase patterns of *E*_*z*_ with 0.6 THz at 12 mm as well as their longitudinal evolutions on the cross-section of y = 0 mm, respectively. (**c**,**d**) Show the corresponding simulated amplitude and phase distributions on the x-y and x-z planes. (**e**,**f**) Give the amplitude and phase profiles extracted from the experiment and simulation results at z = 2 mm, 12 mm, and 24 mm.

Because the convergence of the THz Bessel beam is not a tight-focusing process, the polarization is not influenced in the propagation of the THz wave^19^. Therefore, only *E*_*x*_ and *E*_*z*_ components are measured in the experiment for the generated x-polarized THz Bessel beam. The amplitude distribution of *E*_*z*_ at z = 12 mm and its longitudinal cross section on the x-z plane (y = 0 mm) for the 0.6 THz spectral component are exhibited in Fig. 3a. For the axicon with a 30° opening angle, the ratio between *E*_*z*_ and *E*_*x*_ of the generated THz Bessel beam is about 18%. The *E*_*z*_ component manifests a bilateral symmetric amplitude distribution, which possesses a central zero-amplitude zone, two off-axis main peaks at |x| = 0.5 mm, and bilayer crescent side-lobes at |x| = 1.5 mm and 2.3 mm. Its whole amplitude pattern presents similar characteristics of a dipole radiation, which is analogous to the *E*_*z*_ component of a converging Gaussian beam. The symmetric property of *E*_*z*_ is ascribed to the rotational symmetry breaking of the THz polarization after the axicon^16^. After passing through the axicon, the transmitted THz field has the *E*_*x*_ and *E*_*z*_ components on the horizontal meridian plane, but it only has the *E*_*x*_ component on the vertical sagittal plane. On the longitudinal cross section, the *E*_*z*_ component also manifests a non-diffractive feature as *E*_*x*_ in an approximate propagation distance of 24 mm. The transverse amplitude profiles at z = 2 mm, 12 mm, and 24 mm are extracted, as shown in Fig. 3e. It can be seen that the positions of the two main peaks and outer side-lobes are not sensitive to the propagation distance, although their amplitudes have a variance of less than 30%. The transverse wrapped phase distribution of *E*_*z*_ with 0.6 THz at z = 12 mm and its longitudinal evolution on the cross section of y = 0 mm are shown in Fig. 3b, respectively. The phase of *E*_*z*_ is composed of several half-ring regions which anti-symmetrically locate on both sides of the y axis (x = 0 mm). The phase undergoes a jump of π on the frontier between each adjacent half-ring regions, which gives rise to the amplitude characteristics of the *E*_*z*_ component. On the longitudinal cross section, the phase jump of π always exists on the optical axis with increasing the propagation distance, which implies that two *E*_*z*_ components on the left and right sides of the y-z plane (x = 0 mm) oppositely travel and form the destructive interference on the optical axis in the propagation of the THz Bessel beam. In addition, the phases on the regions of two main peaks separately exhibit a linear variation along the z axis. The phase evolution of *E*_*z*_ also manifests the interference effect of the THz waves refracted by the axicon on different positions of the optical axis.

By utilizing the vectorial Rayleigh diffraction integral, the *E*_*z*_ component is simulated and its complex field can be expressed as^17,18^

6 $$\begin{array}{rcl}{E}_{z}(\rho ,\phi ,z) & = & \frac{j\pi \exp (jkr)\cos \,\phi }{\lambda {r}^{2}}\\  &  & \times {\int }_{0}^{l}d{\rho }_{0}\exp (\mu ){\rho }_{0}[({t}_{p}\,\cos \,\theta +{t}_{s})\rho {J}_{0}(\eta )-2j{t}_{p}{\rho }_{0}\,\cos \,\theta {J}_{1}(\eta )-\,({t}_{p}\,\cos \,\theta -{t}_{s})\rho {J}_{2}(\eta )],\end{array}$$

where ${J}_{1}(\eta )$ is the Bessel function of the first kind with the order 1. Figure 3c and d give the simulated amplitude and phase distributions of *E*_*z*_ with 0.6 THz at 12 mm as well as their evolutions on the longitudinal cross section. The simulated amplitude profiles at z = 2 mm, 12 mm, and 24 mm are extracted and plotted in Fig. 3e, which show a deviation of less than 12% with respect to the experimental results. The experimental and simulated transverse phase profiles at z = 2 mm, 12 mm, and 24 mm are also extracted from Fig. 3b and d, as shown in Fig. 3f. It can be seen that the experiment and simulation results greatly overlap with each other except for a few abrupt phase jumps on the experimental curves.

**Figure 4:**
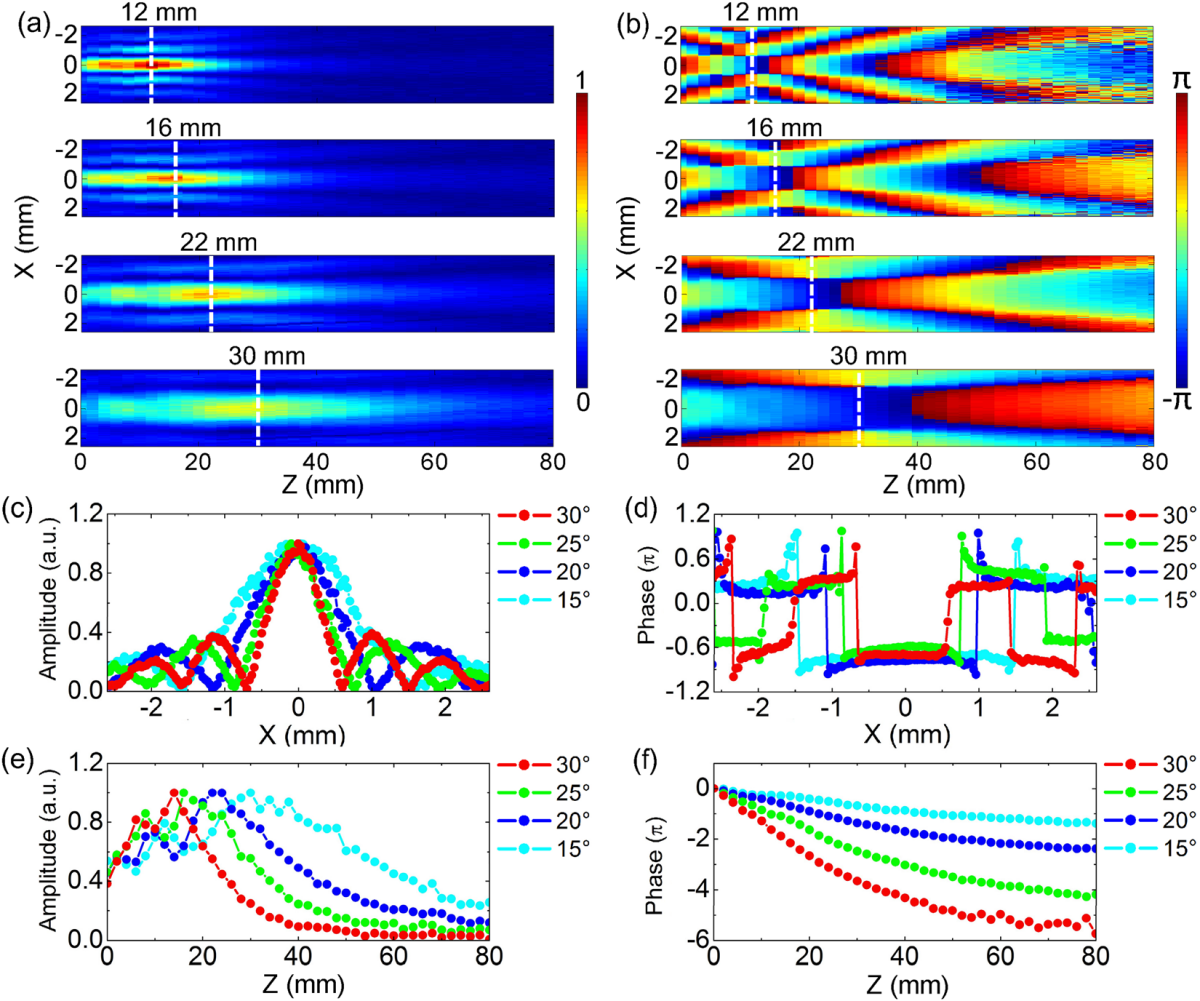
Comparison of the *E*_*x*_ components for THz Bessel beams generated by Teflon axicons with different opening angles. (**a**,**b**) Show the amplitude and phase distributions of *E*_*x*_ at 0.6 THz on the longitudinal cross-section of y = 0 mm for THz Bessel beams generated by axicons with opening angles *α* = 30°, 25°, 20°, 15°. (**c**,**d**) Show the transverse amplitude and phase profiles with *α* = 30°, 25°, 20°, 15° at z = 12 mm, 16 mm, 22 mm, 30 mm. The white dashed lines indicate their positions in (**a**,**b**). (**e**,**f**) Give the longitudinal amplitude and unwrapped phase profiles with different *α* along the optical axis.

To compare propagation characteristics of THz Bessel beams generated by axicons with different *α*, the axicons with *α* = 30°, 25°, 20°, 15° are measured with a 80 mm scan range and a 2 mm scan resolution. Figure 4a shows these amplitude patterns of *E*_*x*_ at 0.6 THz on the longitudinal cross-section of y = 0 mm, respectively. It is obvious that the intensity of the *E*_*x*_ component gradually attenuates with reducing *α*, because the converging process of a generated THz Bessel beam is smoother by using an axicon with a smaller opening angle. To more intuitively compare the focal spot sizes of these THz Bessel beams, the normalized transverse amplitude profiles corresponding to *α* = 30°, 25°, 20°, 15° at z = 12 mm, 16 mm, 22 mm, 30 mm are extracted and plotted together in Fig. 4c. Their positions are marked by white dashed lines in Fig. 4a. The experimental results show that the diameters of these central peaks are 1.4 mm, 1.8 mm, 2.4 mm, 3.2 mm, respectively. The normalized longitudinal amplitude profiles of these THz Bessel beams along the optical axis are also extracted and exhibited in Fig. 4e. The experimental results present that the diffraction-free ranges with *α* = 30°, 25°, 20°, 15° are 24 mm, 32 mm, 42 mm, 59 mm, respectively. Actually, there are some oscillations on the longitudinal amplitude profile curves. The deviations between the THz beams and a prefect Bessel function may arise from the finite apertures or unsharp tips of these axicons^20^. By utilizing Eqs (1) and (2), the diameters of these central peaks and diffraction-free ranges are calculated as 2 *R* = 1.55 mm, 1.93 mm, 2.47 mm, 3.35 mm and *Z*_*max*_ = 25.3 mm, 32.8 mm, 43.3 mm, 59.9 mm for THz Bessel beams with *α* = 30°, 25°, 20°, 15°, which are basically consistent with the experimental phenomena. The longitudinal phase distributions of *E*_*x*_ at 0.6 THz are also compared on the x-z plane for the THz Bessel beams with different *α*, as shown in Fig. 4b. It is apparent that the phase evolution of the THz Bessel beam with a smaller *α* is slower in the propagation process. The transverse phase profiles with *α* = 30°, 25°, 20°, 15° at z = 12 mm, 16 mm, 22 mm, 30 mm are extracted and compared in Fig. 4d, which manifest a similar distribution characteristics. With reducing the value of *α*, the phase profile is gradually stretched along the x axis and the size of the central flat region is progressively magnified. The longitudinal phase profiles along the optical axis are also extracted and unwrapped for comparing the Gouy shifts of the THz Bessel beams with different *α*, as shown in Fig. 4f. The original points of these phase profiles are set as 0 for clarity. It can be seen that the phase variation is almost linear in the non-diffractive region of each phase profile curve, which accords with a previous report^21^. Total phase shifts with *α* = 30°, 25°, 20°, 15° along the z axis are 5.7π, 4.2π, 2.4π, 1.4π, which are shrunk with reducing *α* due to weakening the transverse spatial confinement to the THz field^22^.

**Figure 5:**
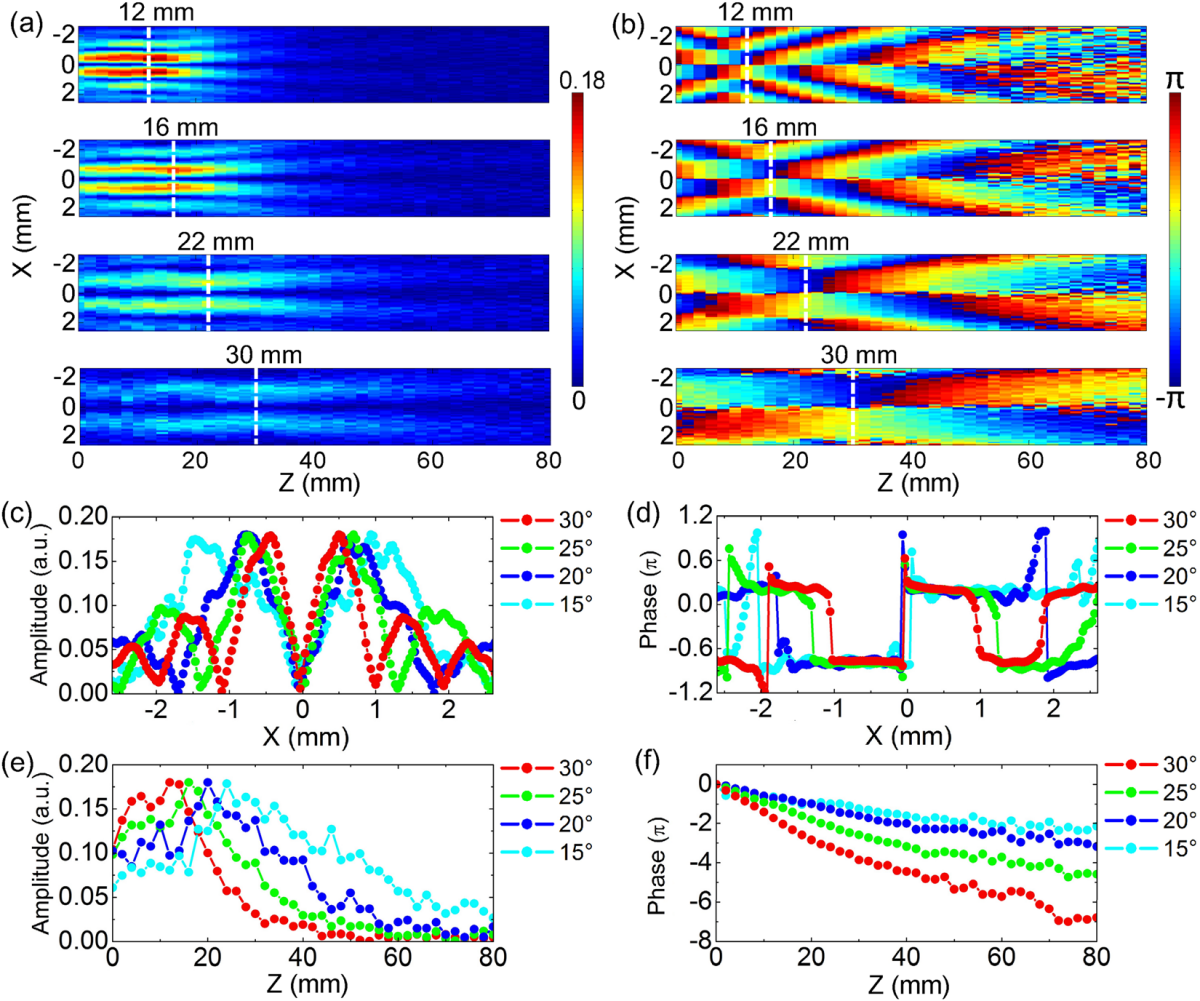
Comparison of the *E*_*z*_ components for THz Bessel beams generated by Teflon axicons with different opening angles. (**a**,**b**) Exhibit the amplitude and phase distributions of *E*_*z*_ at 0.6 THz on the x-z plane for THz Bessel beams generated by axicons with opening angles *α* = 30°, 25°, 20°, 15°. (**c**,**d**) Present the transverse amplitude and phase profiles with *α* = 30°, 25°, 20°, 15° at z = 12 mm, 16 mm, 22 mm, 30 mm. (**e**,**f**) Give the corresponding longitudinal amplitude and unwrapped phase profiles with different *α*.

Similarly to *E*_*x*_, propagation characteristics of the *E*_*z*_ components are also compared for THz Bessel beams generated by axicons with different *α*. Figure 5a exhibits the longitudinal amplitude patterns with *α* = 30°, 25°, 20°, 15° at 0.6 THz on the x-z plane (y = 0 mm). The experimental results manifest that the intensity of *E*_*z*_ is gradually attenuated with reducing *α*. At the same time, the focal spot size and the non-diffractive zone of the THz Bessel beam are increasingly enlarged. The normalized transverse amplitude profiles with *α* = 30°, 25°, 20°, 15° at z = 12 mm, 16 mm, 22 mm, 30 mm are extracted from Fig. 5a and are compared in Fig. 5c. Corresponding to *α* = 30°, 25°, 20°, 15°, the positions of two off-axis main peaks are |x| = 0.5 mm, 0.6 mm, 0.8 mm, 1.1 mm, respectively. Figure 5e show the normalized longitudinal amplitude profiles along the z axis, which shows that the diffraction-free ranges of *E*_*z*_ with *α* = 30°, 25°, 20°, 15° are 22 mm, 31 mm, 41 mm, 57 mm, respectively. In Fig. 5b, the longitudinal phase patterns with *α* = 30°, 25°, 20°, 15° are exhibited on the x-z plane, which present that the phase distribution of *E*_*z*_ is obviously stretched along the transverse and longitudinal directions with reducing *α*. Figure 5d shows the transverse phase profiles with *α* = 30°, 25°, 20°, 15°, which are extracted at z = 12 mm, 16 mm, 22 mm, 30 mm from Fig. 5b. These curves more intuitively present the evolution tendency of the phases for the *E*_*z*_ components with different *α*. In addition, the unwrapped longitudinal phase profiles with different *α* are also extracted on the region of each upper off-axis main peak, as shown in Fig. 5f. The Gouy phase shifts of the *E*_*z*_ components with *α* = 30°, 25°, 20°, 15° are 6.8π, 4.5π, 3.2π, 2.1π, respectively. In general, above analyses to *E*_*x*_ and *E*_*z*_ illustrate that when an axicon with a bigger opening angle is employed, larger transverse wave vectors are introduced and the generated THz Bessel beam possesses a smaller focal spot as well as a shorter non-diffractive zone.

### Vectorial properties of circularly polarized THz Bessel beams

**Figure 6:**
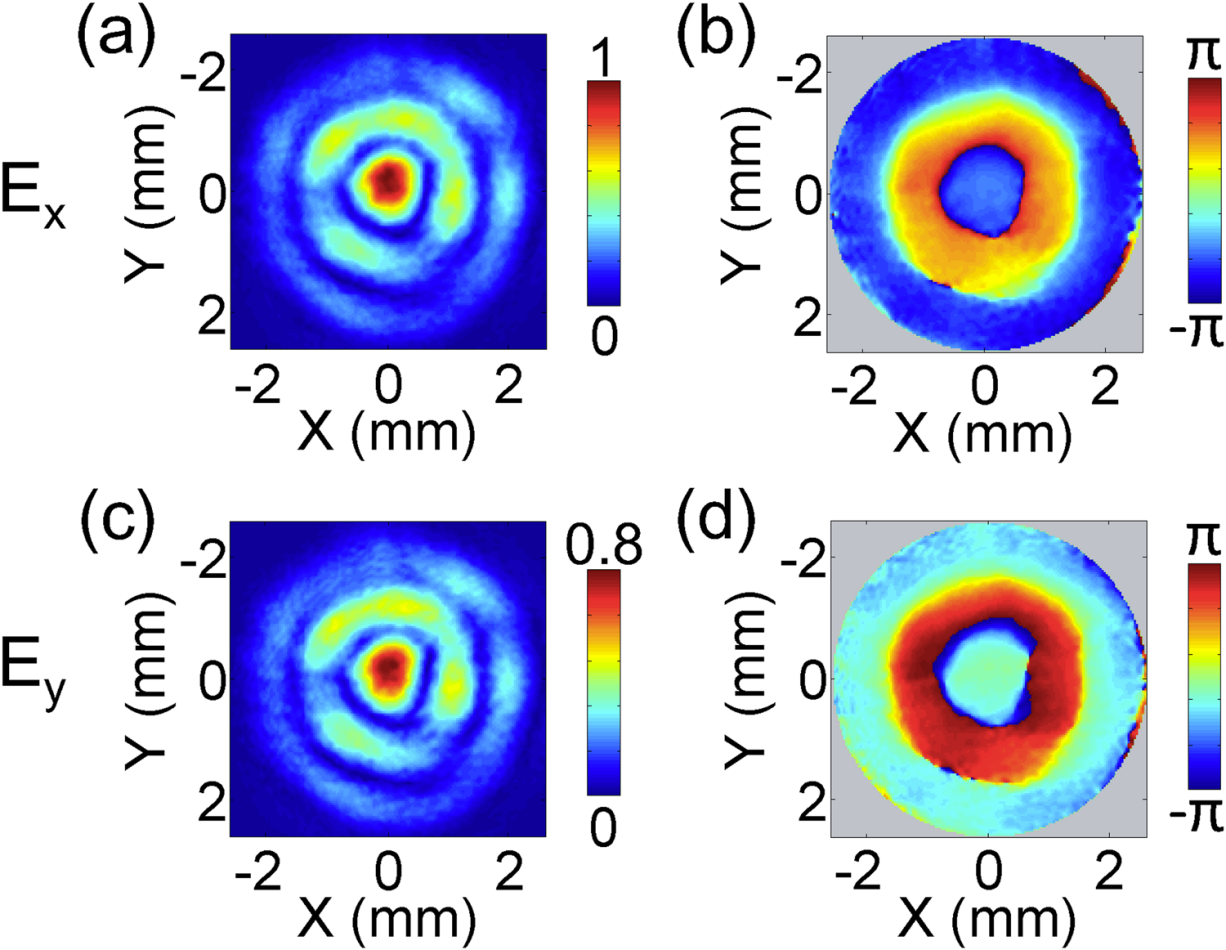
Complex field distributions of the *E*_*x*_ and *E*_*y*_ components for a circularly polarized THz Bessel beam generated by a TQWP and a Teflon axicon with *α* = 30°. (**a**,**b**) Give the amplitude and phase distributions of *E*_*x*_ at 0.6 THz on the focal spot of the THz Bessel beam. (**c**,**d**) Exhibit the corresponding amplitude and phase patterns of *E*_*y*_.

In our previous report^16^, vectorial characteristics of a focusing THz Gaussian beam with a circular polarization are observed, which exhibit a unique vortex distribution of the *E*_*z*_ component. In this work, the vectorial properties of circularly polarized THz Bessel beams are also measured and analyzed. Firstly, a THz Bessel beam with a right circular polarization is generated by utilizing the TQWP and an axicon with *α* = 30°. Its field distributions of the transverse (*E*_*x*_, *E*_*y*_) and longitudinal (*E*_*z*_) components are coherently measured by using the THz digital holographic imaging system. Figure 6 shows the amplitude and phase patterns of *E*_*x*_ and *E*_*y*_ at 0.6 THz on the focal spot, which are similar to those of a linearly polarized THz Bessel beam. Their amplitudes show a circular main peak with a 1.4 mm diameter and two ring-shaped side-lobes with 2.2 mm and 4.0 mm diameters, as shown in Fig. 6a and c. Their phase patterns consist of several concentric circular regions, as shown in Fig. 6b and d. There is a phase shift of π between neighbor circular regions. At the same time, a phase difference of π/2 exists between *E*_*x*_ and *E*_*y*_. In addition, it should be noted that the amplitude difference between the *E*_*x*_ and *E*_*y*_ components is approximately 20% due to the imperfection of the TQWP.

**Figure 7:**
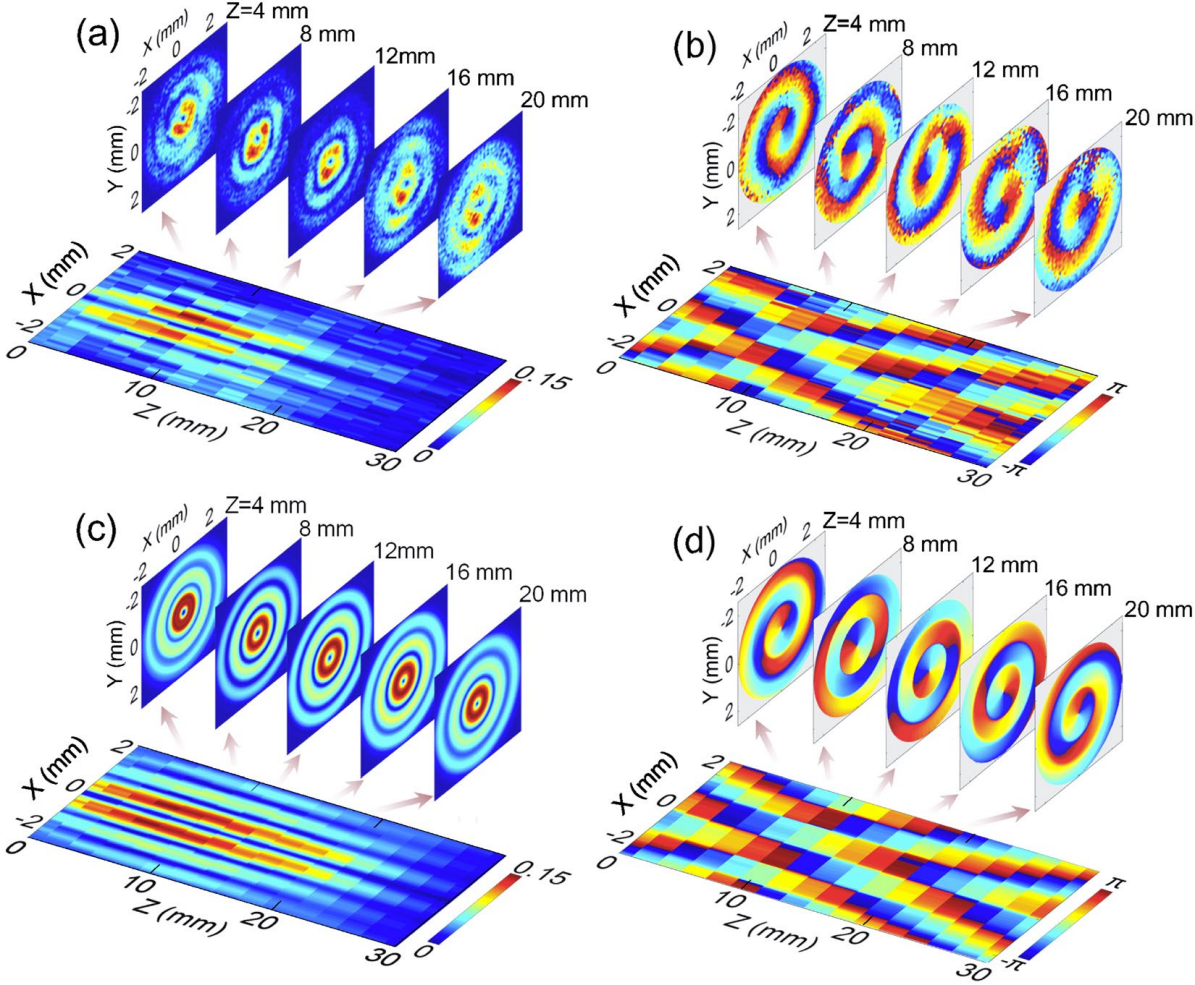
Complex field distributions of the *E*_*z*_ component at 0.6 THz for a circularly polarized THz Bessel beam. (**a**,**b**) Give the amplitude and phase patterns at the propagation distances of z = 4 mm, 8 mm, 12 mm, 16 mm, 20 mm as well as the corresponding longitudinal evolutions on the x-z plane. (**c**,**d**) Show the simulated amplitude and phase distributions on the transverse and longitudinal cross sections.

In this work, the observation to *E*_*z*_ is an interesting point for the circularly polarized THz Bessel beam. By performing the Z-scan measurement, the propagation mode of the *E*_*z*_ component at 0.6 THz is recorded with a 30 mm scan range and a 2 mm scan resolution. Its normalized amplitude as well as wrapped phase distributions at z = 4 mm, 8 mm, 12 mm, 16 mm, 20 mm and the corresponding longitudinal cross sections are presented in Fig. 7a and b, respectively. The *E*_*z*_ component manifests distribution characteristics of an optical vortex. Its amplitude exhibits a typical doughnut shape with a 0.9 mm diameter and annular side-lobes. Here, the diameter is defined as the distance between two amplitude maximums on the light ring along the x axis. The inhomogeneous intensities of these light rings are caused by the imperfect circular polarization of the THz beam. With increasing the propagation distance, the amplitude of *E*_*z*_ also shows an obvious non-diffractive feature. It can be seen that the central light ring almost keeps the same size at different transverse cross sections. The amplitude distribution on the longitudinal cross-section (y = 0 mm) exhibits a diffraction-free range of 22 mm, which is analogous to the case of the linearly polarized THz Bessel beam in Fig. 3a. The phase of *E*_*z*_ exhibits a spiral modality and always rotates along an anticlockwise direction when the THz beam passes through the non-diffractive zone. On the region of the main light ring, the central vortex forms a circular contour profile and its size almost keeps invariant in the propagation distance of 22 mm due to the non-diffractive feature. On the region of the annular side-lobes, the twist direction of the spiral phase appears a reverse during passing through the non-diffractive zone, which is somewhat similar to the phase evolution of the *E*_*z*_ component of a focusing THz Gaussian beam with a circular polarization^16^. The longitudinal phase distribution on the x-z plane is almost the same as the case with a linear polarization in Fig. 3b. In the propagation distance of 30 mm, the Gouy phase shift of *E*_*z*_ is approximately 3.7π. The field distribution of the *E*_*z*_ component is readily simulated for a circularly polarized THz Bessel beam, because it can be considered as a linear superposition of longitudinal fields *E*_*xz*_ and *E*_*yz*_ of two THz Bessel beams with orthogonal linear polarizations. Therefore, the *E*_*z*_ component with a right circular polarization can be expressed as

7 $${E}_{z}={E}_{xz}+{E}_{yz}\exp (j\frac{\pi }{2})$$

The simulated amplitude and phase patterns are given in Fig. 7c and d, which are in good agreement with the experiment results.

**Figure 8:**
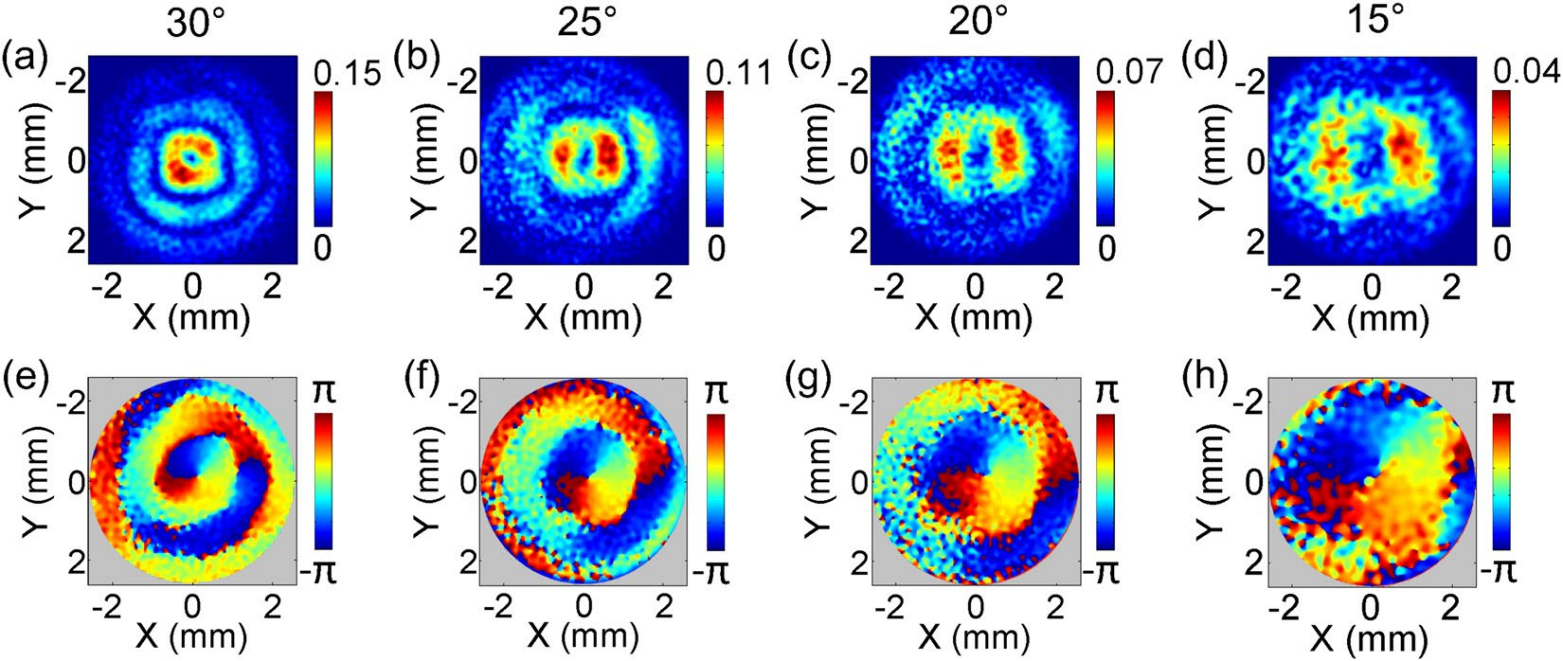
Comparison of the *E*_*z*_ components for circularly polarized THz Bessel beams with different *α*. (**a**–**d**) Give the amplitude distributions of *E*_*z*_ on the focal spots of THz Bessel beams with *α* = 30°, 25°, 20°, 15°. (**e**–**h**) Show the corresponding phase patterns.

Finally, other right circularly polarized THz Bessel beams are generated by using the TQWP and another three axicons. The amplitude and phase distributions of the *E*_*z*_ components with *α* = 30°, 25°, 20°, 15° at 0.6 THz are exhibited and compared on their focal spots, as shown in Fig. 8. All of the *E*_*z*_ components present modalities of optical vortices, including annular amplitudes and spiral phases. With reducing the opening angle, the sizes of the main light ring and central vortex phase region are gradually magnified. The diameters of these main light rings with *α* = 30°, 25°, 20°, 15° are 0.9 mm, 1.2 mm, 1.5 mm, 2.2 mm, respectively. At the same time, the THz intensity progressively weakens because the converging process of the THz Bessel beam with a smaller *α* is smoother. The non-diffractive features of these THz Bessel beams are not repeatedly discussed at here, because their properties are almost the same as the corresponding cases with a linear polarization in Fig. 5a and b.

Here, according to the phenomena obtained in this work and our previous reports^16,23^, we attempt to summarize a general law of the *E*_*z*_ component for a converging optical beam. If a used optical element possesses the axial symmetrical amplitude and phase modulations to the incident wave, such as a spherical lens or an axicon, the *E*_*z*_ component with a linear polarization will present a double-lobe feature and *E*_*z*_ with a circular polarization will manifest the modality of an optical vortex. However, the *E*_*z*_ component will not follow the general law and show a complicated pattern when a non-axial symmetrical modulator is mounted on the optical path, such as a vortex phase plate. The confirmation of the general law needs a rigorous derivation. We will focus on the point in the future work.

## Conclusions

In conclusion, linearly and circularly polarized zero-order THz Bessel beams are generated by utilizing a TQWP and Teflon axicons with different opening angles. In the diffraction-free propagation regions of the THz Bessel beams, their vectorial properties are systematically measured and analyzed by using a THz digital holographic imaging system, including the transverse (*E*_*x*_, *E*_*y*_) and longitudinal (*E*_*z*_) electric fields. For a linearly polarized THz Bessel beam, the *E*_*x*_ component shows concentric circular amplitude and phase patterns with an axial symmetry. The *E*_*z*_ component with a linear polarization exhibits a dipole-like amplitude distribution and a bilateral anti-symmetric phase pattern. The vectorial Rayleigh diffraction integral is used to accurately reproduce the experimental phenomena. By operating Z-scan measurements, the non-diffractive features of *E*_*x*_ and *E*_*z*_ components with different opening angles are measured and compared. With reducing the opening angle of an axicon, the generated THz Bessel beam exhibits a weaker intensity, a bigger focal spot size, a longer diffraction-free range, and a smaller Gouy phase shift. For a circularly polarized THz Bessel beam, the *E*_*z*_ component shows a vortex mode, including an annular amplitude and a spiral phase. In addition, the sizes of the focal spot and central vortex phase of *E*_*z*_ gradually enlarge with reducing the opening angle of an axicon. The novelty of this work is to provide a comprehensive view for exactly understanding vectorial characteristics of a THz Bessel beam in its non-diffractive zone. Particularly, the longitudinal field (*E*_*z*_) of a THz Bessel beam is accurately characterized. We believe that *E*_*z*_ will play a crucial role in the future THz techniques. For instance, *E*_*z*_ of a THz Bessel beam can accelerate or manipulate electrons in a longer diffraction-free range; *E*_*z*_ of a radially polarized THz Bessel beam can provide a smaller focal point than *E*_*x*_ in its non-diffractive zone to enhance the measurement accuracy of a THz imaging system. Therefore, we consider that this work is valuable for developments of THz imaging, THz communications, and micro-particle manipulation. Moreover, the regularities that are acquired in this work can be directly transplanted to investigations of Bessel beams in the visible light.

## Methods

Figure 1a shows the experimental setup of the THz digital holographic imaging system, which is used to characterize vectorial properties of zero-order THz Bessel beams. A Spectra-Physics femtosecond laser amplifier is chosen as the light source, which possesses an 800 nm central wavelength, a 50 fs pulse duration, a 1 kHz repetition ratio, and an 800 mW average power. The output laser is divided into the pump and probe beams for exciting and detecting the THz wave. In the path of the pump beam, the laser pulse of a 790 mW average power is expanded by a concave lens (L1) with a 50 mm focal length and then impinges onto a <110> ZnTe crystal with a 3 mm thickness to launch the THz radiation due to the optical rectification^24^. The THz wave is collimated by a metallic parabolic mirror (PM) with a 100 focal length and the diameter of the THz beam approaches approximately 14 mm. A Teflon axicon is inserted in the path of the THz beam to form a zero-order THz Bessel beam with linear polarization. The out-going THz beam is coherently measured by a detection crystal. The probe beam of a 10 mW average power successively passes through a half wave plate (HWP) and a polarizer (P) for adjusting the probe polarization. A 50/50 non-polarizing beam splitter is employed to reflect the probe beam onto the detection crystal. In the detection crystal, the probe polarization is modulated by the THz field to carry the two-dimensional THz information due to the Pockels effect. The reflected probe beam is acquired by an imaging module, including a quarter wave plate (QWP), a Wollaston prism (PBS), two lenses (L2 and L3), and a CCD camera with a 4 Hz frame ratio. A THz image is extracted based on a balanced electro-optics detection technique^25^. A mechanical chopper is inserted into the pump beam and is synchronously controlled with the CCD camera to remove the background intensity of the probe beam based on a dynamics subtraction method^26^. In this system, the effective imaging area is 5.2 mm × 5.2 mm and the size of a pixel is 32 μm × 32 μm. A series of temporal THz images are captured by sequentially adjusting the optical path difference between the THz and probe beams. At each time delay, 16 frames are averaged to enhance the signal-to-noise ratio and the whole time window is 17 ps. The amplitude and phase distributions of each spectral component are extracted by operating the Fourier transformation to THz images in the time domain.

To analyze vectorial properties of THz Bessel beams, the horizontal, vertical, and longitudinal polarization components (*E*_*x*_, *E*_*y*_, and *E*_*z*_) of a THz field need to be separately measured. In the experiment, a <110> ZnTe crystal with a 1 mm thickness is picked to measure *E*_*x*_ and *E*_*y*_ by varying the probe polarization. By adjusting the HWP and P in the path of the probe beam, the *E*_*x*_ and *E*_*y*_ components can be extracted independently when the angle between the probe polarization and the <001> axis of the ZnTe crystal is 0° and 45°^15^. A <100> ZnTe crystal with a 1 mm thickness is applied to measure *E*_*z*_ and its <010> axis is tuned to 45° with respect to the probe polarization for optimizing the detection efficiency^27^. It should be also noted that both ZnTe crystals have the same measurement sensitivities to a THz field.

To observe the propagation characteristics of THz Bessel beams with linear and circular polarizations, a quartz THz quarter wave plate (TQWP, TYDEX Company, Ruissa) is utilized to translate the THz polarization. The TQWP possesses a 500 μm central wavelength and a 200 μm bandwidth. To record the evolution processes of THz Bessel beams, the TQWP and an axicon are mounted on a linear translation stage and a Z-scan measurement is manipulated. In this work, four commercial Teflon axicons with opening angles of 30°, 25°, 20°, 15° and an aperture diameter of 19 mm are used to generate THz Bessel beams with different non-diffractive zones.
